# Quantitative Methods to Assess Differential Susceptibility of *Arabidopsis thaliana* Natural Accessions to *Dickeya dadantii*

**DOI:** 10.3389/fpls.2017.00394

**Published:** 2017-03-28

**Authors:** Martine Rigault, Amélie Buellet, Céline Masclaux-Daubresse, Mathilde Fagard, Fabien Chardon, Alia Dellagi

**Affiliations:** Institut Jean-Pierre Bourgin, UMR INRA- AgroParisTech 1318, ERL CNRS 3559, Saclay Plant SciencesVersailles, France

**Keywords:** natural variation, *Arabidopsis thaliana*, Dickeya dadantii, quantification, qPCR

## Abstract

Among the most devastating bacterial diseases of plants, *soft rot* provoked by *Dickeya* spp. cause crop yield losses on a large range of species with potato being the most economically important. The use of antibiotics being prohibited in most countries in the field, identifying tolerance genes is expected to be one of the most effective alternate disease control approaches. A prerequisite for the identification of tolerance genes is to develop robust disease quantification methods and to identify tolerant plant genotypes. In this work, we investigate the feasibility of the exploitation of *Arabidopsis thaliana* natural variation to find tolerant genotypes and to develop robust quantification methods. We compared different quantification methods that score either symptom development or bacterial populations *in planta*. An easy to set up and reliable bacterial quantification method based on qPCR amplification of bacterial DNA was validated. This study demonstrates that it is possible to conduct a robust phenotyping of soft rot disease, and that Arabidopsis natural accessions are a relevant source of tolerance genes.

## Introduction

Plants are exposed to biotic and abiotic stresses that lead to important crop yield losses. Considerable advances have been made recently that allow a better understanding of the mechanisms underlying plant–pathogen interactions which determine disease severity at the end. On the one hand, pathogens deploy various virulence systems to invade the plant tissues either by killing cells or by suppressing plant defenses. On the other hand, plants use different defense strategies to counteract this invasion (Dangl et al., 2013). Characterizing the main processes involved in virulence on the pathogen side and involved in defense on the plant side requires a reliable system to score disease severity parameters (Brouwer et al., 2003; Trontin et al., 2011). Indeed, new sequencing technologies and phenotyping open the way to investigate plant genetic determinants of resistance/tolerance to pathogens, but strictly depend on robust quantification methods (Mutka and Bart, 2015). Quantification of disease can rely on different parameters that vary according to the pathogen considered. In most cases, symptom severity is considered as a good indicator of the pathogen’s impact on its host during disease. Another parameter that can be considered, is the *in planta* pathogen growth or pathogen burden (Brouwer et al., 2003). Monitoring the pathogen burden allows a better understanding of the mechanisms controlling disease impact on host. Indeed, plants can be tolerant or resistant to a certain pathogen. Resistance consists of reduced disease severity due to pathogen growth restriction, and relies on specific recognition of pathogen strains via the product of a resistance (R) gene. In the majority of the cases, R genes contain two conserved domains: nucleotide binding domain (NB) and a leucine rich repeat domain (LRR) (St Clair, 2010). Tolerance consists in limiting detrimental effects of disease without a strong reduction of pathogen burden. The underlying mechanisms of tolerance are not specific and mobilize different immunity processes. The outcomes of tolerance or resistance on pathogen populations are different. Resistance can lead to pathogen eradication, but increases the risks of emergence of new hyper-virulent strains. In contrast, tolerance does not reduce pathogen populations and does not favor arms race between hosts and pathogens. Thus, combining quantification of disease severity and pathogen populations *in planta* is crucial.

Bacterial pathogens have the peculiarity of being difficult to control. Indeed, antibiotic use in agriculture is prohibited in most European countries, due to the risk of resistance emergence in the field that could be transferred to clinically important bacteria isolates (Williams-Nguyen et al., 2016). Among the most devastating bacterial diseases, soft rot causes crop yield losses in a large range of species with potato being the most economically important (Czajkowski et al., 2011). Soft rot can be caused by *Dickeya* species or *Pectobacterium* species (Toth et al., 2003; Toth et al., 2011). Bacteria survive in water or in plant organs like potato tubers and infect growing plants in the field (Reverchon et al., 2016). Bacteria multiply on the plant surface then enter apoplastic spaces where they multiply. Typical symptoms are maceration and rotting of infected tissues mainly caused by the massive production of plant cell wall degrading enzymes (PCWDE) that lead to tissue disorganization and then death. Cell wall degradation liberates sugars that are used as carbon source for bacterial cells (Reverchon and Nasser, 2013). The process by which *Dickeya* species infect their hosts corresponds to what is generally described as a necrotrophic lifestyle (van Kan, 2006). Production of these enzymes is tightly controlled at the transcriptional and post transcriptional level in response to environmental factors. Because control of soft rot species is complex, it is a prerequisite to develop a robust and reliable system to score the disease parameters (Czajkowski et al., 2011). For instance, breeding for resistance is hampered by the difficulty to score symptoms and then to correlate this with the bacterial population.

Very few data about plant defense against *Dickeya dadantii* are available (Reverchon et al., 2016). Accumulation of reactive oxygen species is involved in reducing *D. dadantii* infection on *Saintpaulia ionantha* and Arabidopsis (Santos et al., 2001; Reverchon et al., 2002; Fagard et al., 2007). Jasmonic acid is involved in bacterial attraction and defense (Fagard et al., 2007; Antunez-Lamas et al., 2009). Iron homeostasis plays a pivotal role in Arabidopsis defense against *D. dadantii* (Aznar et al., 2014, 2015; Nam Phuong et al., 2012). In order to gain insight into the plant defense arsenal effective against *Dickeya* spp. and to identify tolerance genes, genetic screens can be very powerful. To be able to set up genetic screens, it is necessary to find differentially susceptible plant genotypes by scoring robust quantitative traits. The use of Arabidopsis as a model plant to accelerate discovery of tolerance or resistance genes in crops proved to be efficient in several instances (Piquerez et al., 2014). The rationale of using the model plant Arabidopsis rather than potato is the availability of genetic tools such as fully sequenced genotypes, mutant libraries allowing a rapid identification of candidate genes. In this work, we investigated the feasibility of the exploitation of natural variation in *Arabidopsis thaliana* L. (Arabidopsis) to highlight differential susceptibilities by comparing different quantitative traits. In addition, Arabidopsis is a host for *D. dadantii* displaying a compatible interaction with spreading symptoms (Fagard et al., 2007). In potato, candidate genes involved in tolerance QTLs to *Phytophthora infestans* were found to be involved in tolerance to the oomycete after expression in Arabidopsis (Pajerowska-Mukhtar et al., 2008) indicating that common mechanisms of tolerance do exist in Arabidopsis and potato.

The most studied disease quantitative trait in genetic studies is the visible symptom. Another important trait, is the *in planta* pathogen burden. *In planta* bacterial population estimation by classical counting of bacterial colonies is tedious and time-consuming. To improve bacterial population estimation *in planta*, we designed primers to PCR-amplify bacterial DNA from infected tissues. We have determined the correlation between several disease parameters in different plant genotypes harboring different tolerance levels to *D. dadantii*.

## Materials and Methods

### Plant and Pathogen Material

Wild-type accessions of Arabidopsis Bur-0, Can-0, Col-0, Cvi-0, Edi-0, Ge-0, Oy-0 and Sakata were obtained from the Versailles Arabidopsis Stock Center (INRA Versailles France^1^). Arabidopsis seeds were stratified by incubation for 2 days at 4°C in 0.01% agarose in water (w/v) in the dark, then, were sown in sand. Homogeneous germination occurred 2 days after sowing. Three times per week, the pots were watered (by immersion of their base) in a mineral solution containing 2 mM nitrate or 10 mM nitrate (Supplementary Table 1). The pH of the watering solutions remained between 5.1 and 5.5 (Lemaitre et al., 2008). During the first 2 weeks, they were watered with the 2 mM nitrate containing solution to avoid intoxication with excess nitrate. The four following weeks, they were watered with a solution containing 10 mM nitrate (Supplementary Table 1). Plants were grown under short days (8-h light/16-h dark) with 21°C temperature and 70% relative humidity. Six- week-old plants were heavily watered and covered with a transparent plastic 16 h before inoculation. The cover was kept in place throughout the assay to maintain high-humidity conditions.

### Bacterial Strain and Inoculation Method

The experiments were performed with the *D. dadantii* 3937 strain constitutively expressing a *gfp* fusion and resistant to gentamycin (Asselbergh et al., 2008; Chapelle et al., 2015). Bacteria were grown in Luria-Bertani medium supplemented with 10 μg/mL gentamycin (Sigma). For plant inoculations, a small hole was made with a needle in the leaf, and then, 5 μL of a bacterial suspension at a density of 1 × 10^8^ Colony Forming Unit/mL (CFU) made up in 50 mM potassium phosphate buffer (pH 7) was spotted on the top of the hole. In each experiment, six plants were inoculated for each genotype and three leaves by plant were inoculated. In order to avoid bias linked to symptom severity, leaves were randomly chosen to be processed by each method of bacterial quantification (CFU counting or qPCR).

### Surface Measurement

Photographs of infected plants were taken for each individual leaf. The surface of the lesions were analyzed using the open source software ImageJ https://imagej.nih.gov/ij/.

### Count of Bacterial Colonies

To monitor *in planta* viable bacterial populations, infected leaves were individually harvested in 500 μL 0.9% NaCl. After grinding, the suspension was diluted following a 10-fold dilution series up-to 10^-6^ or 10^-7^ fold. Ten microliters droplets were then plated out on LB medium containing 10 μg/mL gentamycin following the drop-plate method (Herigstad et al., 2001). For each dilution in the series, two 10-μL droplets were placed on LB plates and incubated at 28°C for 48 h. CFUs were counted on plated droplets that contained between 4 and 40 colonies and the number of CFU per leaf was calculated.

### Bacterial DNA Extraction

Bacterial DNA was extracted from pure *D. dadantii* liquid cultures. An overnight LB grown bacterial culture was pelleted by centrifugation. The pellet was suspended in TE(Tris-EDTA) buffer Proteinase K and SDS were added to a final concentration of 100 μg/mL and 0.5%, respectively. The extract was thoroughly mixed and incubated at 37°C for 1 h, then NaCl was added to a final concentration of 0.83 M. To precipitate cell debris, cetyltrimethylammonium bromide (CTAB)/NaCl (10% CTAB, 0.7 M NaCl) was added to reach the final proportion of 8/70th of the volume, then incubated 10 min at 65°C. An equal volume of chloroform/isoamylic alcohol was added, followed by mixing and spinning for 5 min. The supernatant was recovered and subjected to a phenol/chloroform/isoamylic alchohol extraction followed by a precipitation in isopropanol.

### DNA Extraction from Infected Leaves

After harvesting, infected leaves were individually frozen in liquid nitrogen then ground. DNA extraction was performed using the CTAB extraction method. In each tube containing one ground leaf, 400 μL of CTAB buffer (2% cetyltrimethyl-ammonium bromide, 1% polyvinylpyrrolidone, 100 mM Tris-HCl, 1.4 M NaCl, 20 mM EDTA, 0.2% β-mercapto-ethanol) was added. The samples were then incubated 30 min at 60°C. Four hundred microliters of chloroform: isoamylic alcohol (24:1) were then added. The samples were centrifuged for 10 min at 7000 rpm in a micro-centrifuge and 300 μL of the supernatant were harvested, then DNA was precipitated by adding 300 μL of isopropanol. After centrifuging 15 min at 7000 rpm, the pellet was recovered and washed with 70% ethanol then DNA was dissolved in 30 μL of TE buffer.

### Quantitative PCR Amplification of Bacterial DNA

DNA from *D. dadantii* bacterial cultures was used as a standard. A standard curve was obtained by a fivefold serial dilution starting from a concentration of 5 ng/μL. Amplifications of DNA from infected leaves was performed on a 1/50th of the purified DNA (see section above). Primers used are: *PelA*-Forward: 5′-CCGCAACGTCTACATCCAAA-3′; *PelA*-Reverse: 5′- CGTCGCCTTTTTCGTAATGC-3′; *RpoB*-Forward: 5′- AATCGAAGGTTCCGGGATTC -3′; *RpoB*-Reverse: 5′- GCCGTTACGGATATCGATGAG -3′. Two and a half microliters of the 1/50th diluted DNA was subjected to real time qPCR using SYBR Green PCR Mastermix (Eurogentec) and gene-specific primers. Absolute DNA quantities were obtained based on the standard curve of pure bacterial DNA. The DNA content in each leaf was converted into Bacterial cell EqDNA according to Supplementary Table 2 formulas.

## Results

### Symptom Quantification on a Collection of Arabidopsis Natural Accessions

**FIGURE 1:**
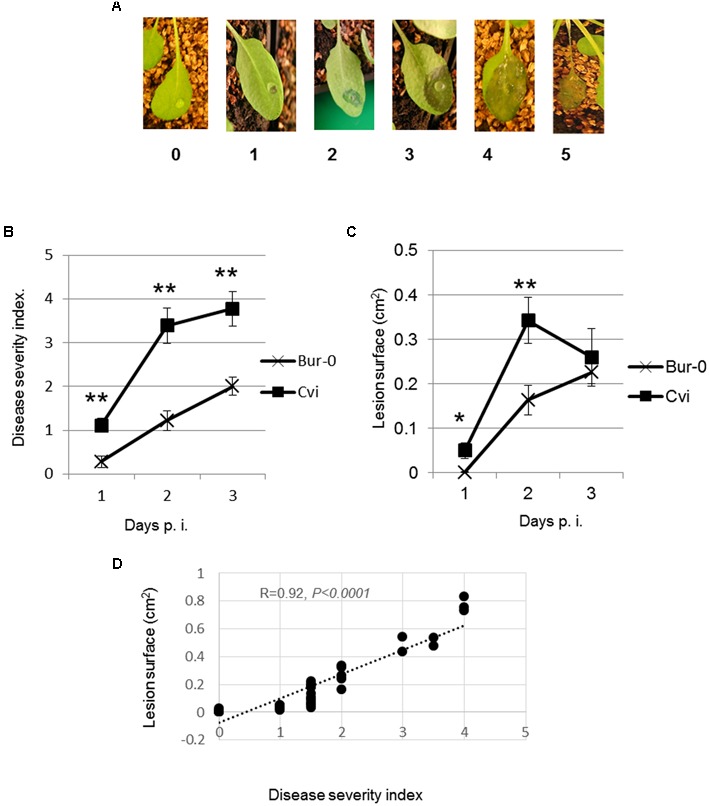
**Correlation between macerated surface and disease severity on Arabidopsis leaves infected with *Dickeya dadantii.* Leaves were inoculated with 5 μL of bacterial suspension. (A)** Image representing stages of the disease severity index scale. **(B)** Leaf symptoms were scored according to the scale of 0–5 (Fagard et al., 2007). **(C)** Photographs were taken at indicated times after inoculation. Diseased areas were measured using the computer program ImageJ. **(D)** Correlation between DSI and LS 48 h p.i. Experiments were repeated at least three times. Data from one representative experiment are shown. Error bars: standard deviation. Mean were compared using Student’s *T*-test. *n* = 10–18. ^∗^*p-*value < 0.05, ^∗∗^*p-*value < 0.001.

Visual symptom scoring is usually performed based on a disease severity scale or based on surface measurement. We compared these two methods for the quantification of visible symptoms caused by *D. dadantii* on different Arabidopsis accessions. The first method consists in attributing a disease severity index (DSI) to each symptom based on the scale indicated in **Figure 1A** as previously described (Fagard et al., 2007). The second method consists in measuring the surface of the lesion hereafter designated as “lesion surface” (LS).

To minimize environmental effects potentially due to soil composition, seedlings were grown on sand and watered with a mineral solution as indicated. Leaves of 6-week-old seedlings were inoculated by making a hole with a needle followed by the application of 5 μL of a bacterial suspension to ensure that each leaf was inoculated with the same number of bacteria. *D. dadantii* infection causes tissue maceration visible 24–48 h post infection (24–48 h p.i.). The maceration spreads starting from the hole. Symptoms are scored during the 1st days (here 3 days) using the 0–5 DSI scale (**Figure 1A**). To compare these two procedures, photographs were taken at different time points after inoculation, areas of maceration were measured by computer-assisted analysis of the images. In order to compare the two methods, we chose Arabidopsis accessions displaying different sensitivities to infection based on symptom scoring (data not shown). These accessions are Bur-0 displaying a reduced sensitivity and Cvi-0 displaying a high sensitivity.

The progression curves of LS and DSI indicated that the relative susceptibilities of the two accessions were the same for both symptom-scoring methods, Bur-0 being the most tolerant, Cvi-0 being the most susceptible. However, significant differences were observed between DSI and LS after 24 and 48 h following infection. Indeed, the DSI progression curve shows a regular increase over 3 days (**Figure 1B**) while the LS started to diminish in Cvi-0 at 48 h p.i. (**Figure 1C**). This reduction could be attributed to the fact that severe maceration causes shrinking and folding of the leaf tissue. Thus, at later infection stages, LS may not correctly reflect the severity of symptoms. We concluded that the best time to highlight different sensitivity levels is at 48 h p.i. We determined the degree of correlation between both quantification methods (DSI and LS). Statistically significant linear correlation was obtained between LS and DSI with a Pearson correlation coefficient of 0.92 (**Figure 1D**). Thus, DSI and LS provide similar estimations of the Arabidopsis genotypes susceptibility to *D. dadantii*.

**FIGURE 2:**
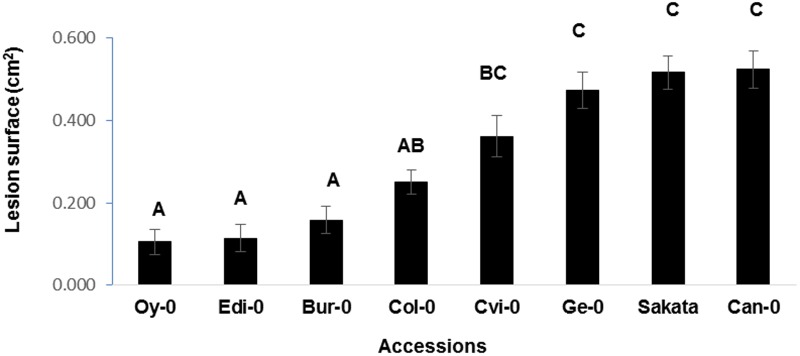
**Quantification of disease on a collection of Arabidopsis accessions.** Disease severity was quantified by LS on the indicated Arabidopsis genotypes 48 h p.i. Experiment was repeated three times with similar results. Representative data are shown. Means with different letters are significantly different at *p* < 0.05 as determined using XLSTAT ANOVA Tukey HSD test.

To determine whether the quantification of disease based on symptom severity can be used to discriminate between *Arabidopsis* genotypes, we studied *D. dadantii* infection on a panel of eight accessions chosen for their different origins (data not shown). For this purpose, bacterial infection was performed on the accessions Bur-0, Can-0, Col-0, Cvi-0, Edi-0, Ge-0, and Oy-0. Disease severity estimated by LS at 48 h p.i. indicates that three statistically distinct groups could be defined. According to the LS criterion, the most tolerant accessions are Oy-0, Edi-0, and Bur-0. The most susceptible ones are Ge-0, Sakata, and Can-0 (**Figure 2**). These data indicate that natural accessions of Arabidopsis display different levels of tolerance to *D. dadantii* suggesting that tolerance associated loci might be found in this plant species.

### Bacterial Quantification *In planta* by qPCR Amplification of Bacterial DNA

**FIGURE 3:**
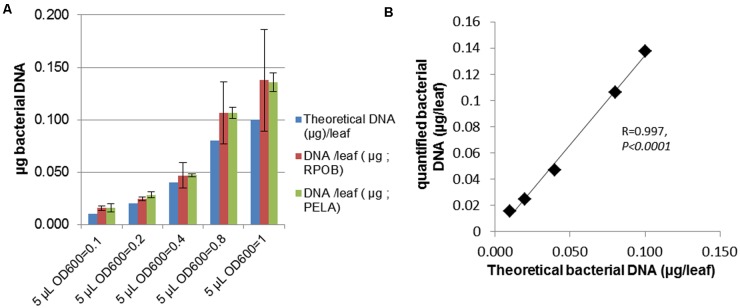
**Bacterial population estimation by qPCR in samples with known bacterial titer. (A)** Leaves were inoculated with 5 μL of a bacterial suspension at increasing concentrations. Leaves were immediately frozen and total DNA extracted then qPCR was performed with primers amplifying *RPOB* or *PELA*. Bacterial DNA quantities were determined based on a standard curve obtained with pure bacterial DNA. **(B)** Estimated bacterial DNA was plotted against expected values to check reliability of the quantification method.

To perform large scale phenotyping of plants, robust and simple methods are needed. We were interested in phenotyping bacterial burden *in planta*, which is a complementary trait to the visible symptom. To investigate *in planta* bacterial populations, serial dilutions of samples followed by plating and colony counting are usually performed (Tornero and Dangl, 2001). However, this method is time-consuming and requires the use of a bacterial strain that harbors a resistance to an antibiotic. We wanted to develop a system to quantify bacteria by qPCR that could be easier to perform on a larger scale. For this purpose, before quantifying unknown bacterial titers, we checked whether DNA amplification allowed us to correctly determine a known bacterial titer *in planta*. To obtain leaves with a known bacterial titer, Arabidopsis leaves were inoculated with different known amounts of bacteria, then were immediately frozen. Total DNA was extracted as described, and two bacterial genes were amplified (*pelA* and *rpoB*) to avoid artifacts related to primer sequence. The *pelA* gene encodes a pectate lyase present in pectinolytic bacteria (Hugouvieux-Cotte-Pattat et al., 2014). The *rpoB* gene encodes an RNA polymerase which is a powerful tool used for bacterial identification (Mollet et al., 1997). These primers were used in a previous study (Nam Phuong et al., 2012), and their specificity was checked using as a template DNA of the closely related *D. solani* (Pedron et al., 2014). Amplification efficiencies with *D. solani* genomic DNA were out of acceptable range thus confirming the specificity of our primers (data not shown). Sequence alignments of *pelA* and *rpoB* primers with the corresponding genes from *D. solani* show several mismatches (Supplementary Figure 1). To quantify *D. dadantii* DNA, a standard curve was obtained using a serial dilution of genomic bacterial DNA from *D. dadantii* 3937. Data on **Figure 3** show that bacterial DNA estimated by qPCR is in agreement with the expected DNA in the inoculum (Supplementary Table 2) based on the fact that a *D. dadantii* bacterial suspension of 0D_600_ = 0.1 contains approximately 5 × 10^8^ CFU/mL (Antunez-Lamas et al., 2009) and that a bacterial cell contains approximately 5 fg of DNA (Hutchison and Venter, 2006). Bacterial DNA amounts estimated via *pelA* and *rpoB* amplification were similar indicating that DNA quantification does not depend on the primers’ sequence. A highly statistically significant correlation was observed between experimentally quantified bacterial DNA via qPCR and theoretically expected bacterial DNA (**Figure 3B**). These data indicate that our qPCR-based quantification method is a reliable tool to estimate bacterial populations *in planta* at the initial stage of infection. Bacterial populations estimated by qPCR will be designated hereafter as “Bacterial cell EqDNA.”

### Individual Sample Analyses of Bacterial Population and Symptom Severity

Quantifying disease based on symptoms or based on bacterial populations provides different biological information. Indeed, it is conceivable that some plants may harbor a high inoculum without displaying symptoms. For instance, *Dickeya* species are known to survive in a latent state in plant tissues without causing symptoms (Toth et al., 2003; Czajkowski et al., 2011, 2015). Thus, we investigated possible correlations between bacterial populations and visible symptoms in individual leaves.

For this purpose, we quantified bacteria *in planta* using two different methods. The first method is the classical count of bacterial colonies after tissue grinding and serial dilutions followed by plating and counting the CFUs which is widely used in the literature (Tornero and Dangl, 2001; Lebeau et al., 2008; Antunez-Lamas et al., 2009). The second method is based on qPCR quantification of bacterial DNA in inoculated plants. We therefore compared these two methods, using plants at the extremes of the gradient of sensitivity of our panel of differentially susceptible accessions to investigate whether symptom severity is related to bacterial populations when disease progression has occurred (24 and 48 h p.i.). Leaves of Cvi-0 and Bur-0 plants were inoculated with 5 μL of a bacterial suspension at 5 × 10^8^ CFU/mL. Leaves displaying different intensities of DSI and of LS were harvested individually. As expected, bacterial populations at 0 h p.i., quantified by both methods corresponded to the inoculum, i.e., 25,000 bacteria/leaf (data not shown). Then, at 24 and 48 h. p.i., the bacterial titers in each inoculated leaf were determined, using either the DNA quantification method or the serial dilution method. By this means, we were able to attribute to each infected leaf 3 parameters: (1) DSI, (2) LS, (3) bacterial titer/leaf either estimated via CFU or via Bacterial cell EqDNA.

**FIGURE 4:**
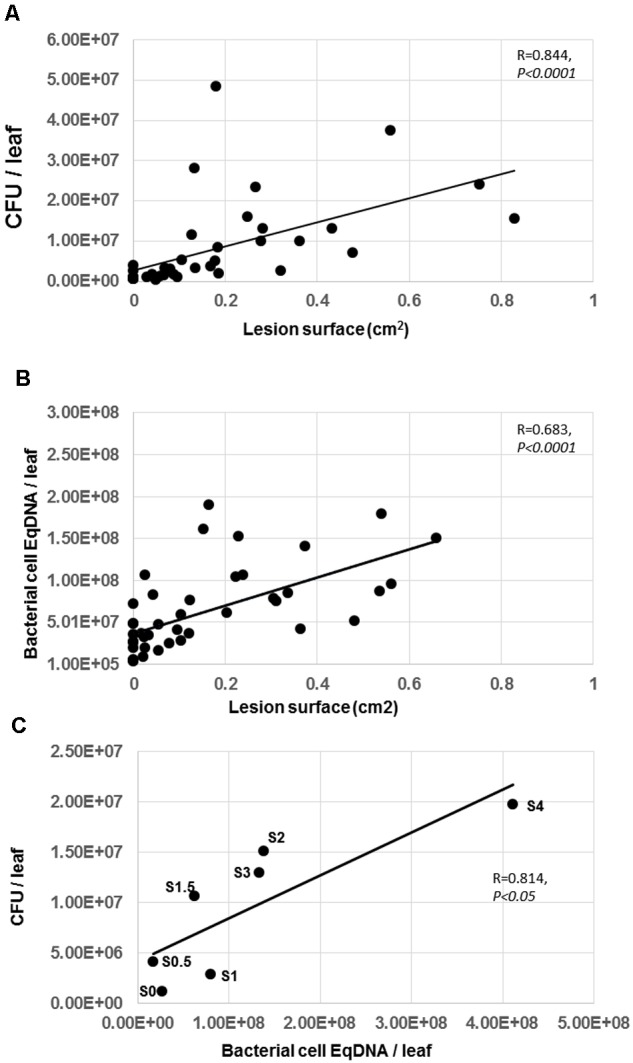
**Correlation between lesion surface and bacterial population estimated by CFU or DNA in Arabidopsis leaves. (A)** Bacterial population estimated by CFU was plotted against LS. **(B)** Bacterial population estimated by qPCR was plotted against LS. **(C)** Bacterial population estimated by qPCR was plotted against bacterial populations estimated by CFU in each class of symptoms (S0 = Stage 0, S0.5 = Stage 0.5, etc). Experiment was repeated twice with similar results. Representative data are shown. Determined coefficients are indicated (Spearman for **A** and **B**, Pearson for **C**).

To determine whether visible symptoms were correlated with bacterial titers, we plotted LS against bacterial titers estimated by CFU or by Bacterial cell EqDNA. **Figures 4A,B** indicate that there was a statistically significant linear correlation between bacterial populations either estimated as CFU or as Bacterial Cell EqDNA and symptom severity (LS). Interestingly, measured bacterial populations were most variable at the beginning of the infection process, corresponding to LS below 0.2 cm^2^ (stages 1–2). Furthermore, we observed that in some cases bacterial populations reached higher levels in leaves with low LS compared to leaves with higher LS, indicating that in some cases bacteria can survive in leaves under a latent state. Taken together, our data show that bacterial populations are positively correlated to disease symptoms. However, bacterial populations *in planta* show, an important variability at the early stages of the infection, which may explain why it is quite complex in some cases to evaluate disease severity with this bacterium (Czajkowski et al., 2011). We note that the estimated bacterial populations by qPCR were in general higher than the bacterial populations estimated by counting of CFU although immediately after the inoculations, the initial inocula were identical. One possible explanation for this difference is that qPCR-based estimation of bacterial cells includes a part of the bacterial populations that died during the infection process and thereby over-estimates the amount of bacteria. Conversely, grinding the infected tissue then diluting and counting growing colonies as CFU probably kills a proportion of the bacterial cells and from the plant defense system.

To compare CFU/leaf and Bacterial Cell EqDNA/leaf, it was not technically possible to have two different estimations of the bacterial populations for the same leaf. To circumvent this problem, we compared the CFU/leaf and Bacterial Cell EqDNA/leaf for leaves displaying the same DSI. Indeed, this parameter could be scored for every leaf. To be more precise in our procedure, we divided the symptom stages as follows: Stage 0, Stage 0.5, Stage 1, Stage 1.5, Stage 2, Stage 2.5, Stage 3, Stage 3.5, Stage 4 (In our data, there was no Stage 5 because we used data for the 24 and 48 h p.i. time-points, when the symptoms rarely reach stage 5). Thus, for each stage, we could determine a mean bacterial population quantified as CFU/leaf and a mean bacterial population as Bacterial Cell EqDNA/leaf. This allowed us to check whether there is a correlation between these two parameters. **Figure 4C** shows that the Pearson correlation coefficient between the two datasets is 0.814 with statistically significant correlation indicating that the two bacterial quantification methods are equivalent. Although CFU and Bacterial cell EqDNA do not derive from the same individuals, this comparison provides an additional indication that both methods are consistent.

### Quantification of Bacterial Populations on a Collection of Arabidopsis Natural Accessions

**FIGURE 5:**
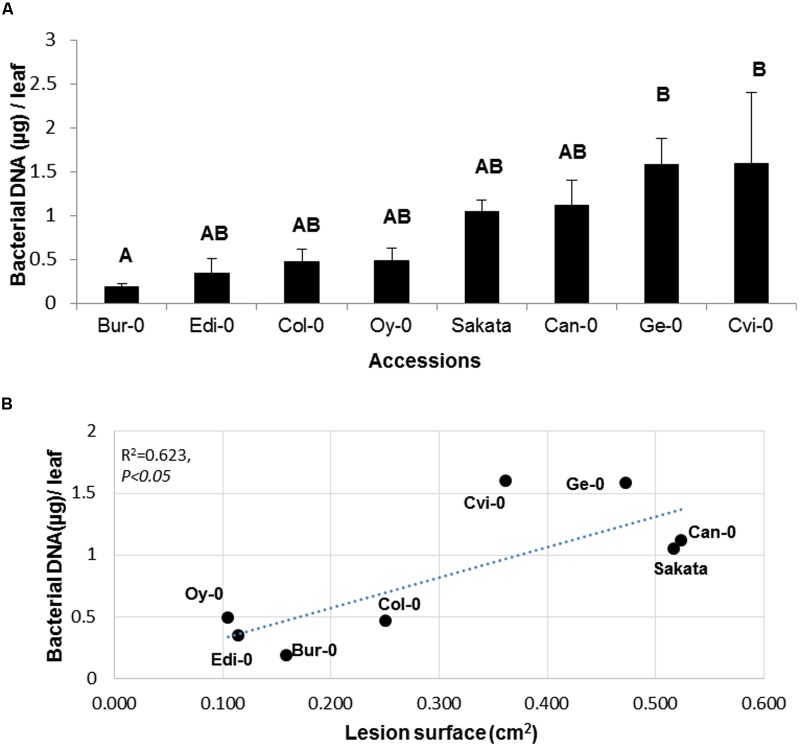
**Quantification of disease and bacterial populations on a collection of Arabidopsis accessions. (A)** Bacterial population estimated by qPCR on the indicated Arabidopsis genotypes 48 h p.i. **(B)** Bacterial population estimated by qPCR was plotted against LS for each genotype. Experiment was repeated at least twice with similar results. Representative data are shown. Means with different letters are significantly different at *p* < 0.05 as determined using XLSTAT ANOVA Tukey HSD test.

To assess whether differential symptom severity on the eight accessions analyzed in **Figure 2** was associated with a differential pathogen growth, bacterial populations were quantified by qPCR in these accessions. This allowed the definition of two statistically distinct groups (**Figure 5**). According to the bacterial population estimated by Bacterial cell EqDNA, the most tolerant accessions are Bur-0 and Edi-0 which are also classified as the most tolerant according to the LS criterion (**Figure 2**). The accessions displaying the highest bacterial population estimated by bacterial cell EqDNA, are Ge-0 and Cvi-0 which are also found in the most susceptible group according to the LS criterion. Disease symptoms as LS were plotted against bacterial populations for each accession to further determine the level of correlation between these two criteria. We found that the LS and bacterial DNA were significantly correlated with a correlation coefficient of 0.62 indicating again that there is an overall consistency between bacterial populations and visible disease symptoms although LS is more discriminant in this experiment. Estimation of bacterial populations provides complementary biological information about the mechanisms underlying the interaction. Taken together, these data show that although, this pathosystem presents a difficulty due to the presence of latent bacterial populations during infection, *Arabidopsis* is a good model to study genetic diversity of tolerance.

## Discussion

Identifying novel genes involved in defense processes against the necrotrophic pathogen *D. dadantii* is very challenging. Indeed, this bacterium causes major economic losses but very few data are available about potential tolerance genes. Taking advantage of the available novel genomics tools and QTL mapping populations in the model plant Arabidopsis opens new perspectives concerning the identification of tolerance genes to this bacterium. The feasibility of such approaches with *D. dadantii* is hampered by the lack of availability of reliable disease quantification methods. We addressed this issue by monitoring different disease parameters in a detailed experimental design. This experimental design was aimed at validating and comparing the different quantification methods in order to allow the choice for their use at larger scales (**Table 1**). We first supported our previously published scoring system by a quantitative method based on measurements of the macerated area. We then showed that quantifying bacterial populations by qPCR *in planta* is a reliable method to phenotype plant susceptibility to *D. dadantii*.

**Table 1:** Comparative analysis of the different methods to quantify Arabidopsis susceptibility levels against *Dickeya dadantii*.

Methods to score susceptibility	Advantages	Drawbacks
DSI	– Simple and quick	- Limited precision
LS	– Precise	– Time consuming
		– Not informative at later infection stages
CFU/leaf	– Informative about bacterial titer	– Tedious and time consuming
	– Cheap	– Underestimates bacterial populations
	– Precise	– Requires the use of a strain resistant to an antibiotic
	– Requires immediate processing
Bacterial Cell EqDNA/leaf	– Informative about bacterial titer	– Costly
	– Simple and quick
	– Frozen tissue can be processed after sampling
	– Precise


Our data and others’ shows that the infection process by *D. dadantii* is quite complex and may result in asymptomatic lesions containing large amounts of bacteria in some cases, and important lesion development with limited bacterial populations in other cases. This asymptomatic phase led to the proposition that soft rot causing bacteria may be considered as hemi-biotrophs (Kraepiel and Barny, 2016). The asymptomatic phase ends at the onset of pectinase production that is under the control of a complex regulatory network in *Dickeya* spp. Signaling networks that sense environmental cues are activated via transcription factors including KdgR, PecS, Fur, and PecT responding to pectin fragments, immune signals, iron and temperature, respectively (Reverchon et al., 2016). In *D. dadantii*, a quorum sensing signal produced via a gene cluster named Vfm; coordinates the production of PCWDE at high bacterial density (Nasser et al., 2013). Notwithstanding the occurrence of asymptomatic infections, we show here that it is possible to obtain reliable estimations of bacterial populations by qPCR that are equivalent to estimations monitored by the classical CFU counting, and that this quantification method is a good indicator of the overall plant susceptibility (based on measurement of symptoms). This is consistent with a recent study showing that *Pseudomonas* sp. quantification *in planta* by qPCR is correlated with counting of bacterial colonies estimated by CFU in previously characterized susceptible Arabidopsis mutants (Ross and Somssich, 2016). In our work, instead of validating the quantification methods on previously characterized susceptible mutants, we were able to uncover significantly different susceptibility groups within a collection of Arabidopsis natural accessions uncharacterized previously following *D. dadantii* infection.

Quantifying DNA as a phenotyping method is a well-known method for fungal pathogens (Klosterman, 2012). Estimation of pathogenic bacteria by qPCR is performed to determine the level of natural contaminations in the field (Czajkowski et al., 2015), but it is not routinely used for pathogenic bacteria in an experimental setting. A previous work showed that it was possible to use qPCR to study bacterial populations *in planta* using *Pseudomonas syringae* and *Pectobacterium carotovorum* as examples (Brouwer et al., 2003), but here we go further to demonstrate the correlation with CFU and disease severity, a prerequisite for the use of this technique in experimental plant pathology. Although quantifying bacterial populations *in planta* based on DNA includes dead bacteria, we were able to show that this does not hamper the significant correlations between CFU bacterial counting and Bacterial Cell EqDNA. Quantifying bacterial RNA by RT-qPCR may allow the quantification of living bacteria. However, this would require the use of a constitutively expressed bacterial gene *in planta*, and would require an additional expensive step, which is the reverse transcription.

For most necrotrophs and hemi-biotrophs infecting a large range of hosts, the genetic determinants of plant immunity rely on tolerance rather than specific resistance (Lai and Mengiste, 2013; Roux et al., 2014). Thus, it is likely that the plant immunity against *D. dadantii* relies on tolerance processes. Because tolerance is a quantitative trait, the availability of powerful quantification methods is crucial. Tolerance is usually controlled by multiple loci and resistance is usually controlled by a single locus encoding a resistance (R) gene (St Clair, 2010). However, some exceptions do exist especially with the example of the bacterial wilt *Ralstonia solanacearum* (Huet, 2014). Tolerance can be conferred by a single R gene like it is the case for the R gene *RRS-1* that confers tolerance in Arabidopsis accession Kil-0 (Van der Linden et al., 2013). Conversely, multiple R genes can be involved in tolerance to bacterial wilt in *Medicago truncatula* (Vailleau et al., 2007).

By the present work, we provide a tool simple to set up in order to phenotype sensitivity of Arabidopsis plant genotypes to the bacterial pathogen *D. dadantii*. This study demonstrates that it is possible to conduct a robust phenotyping of soft rot disease despite the occurrence of latent bacteria, and that Arabidopsis natural accessions are a relevant source of tolerance genes. Thus, it would be of great interest to use our scoring methods to identify tolerance genes either using QTL mapping in a recombinant segregating population or by performing a genome wide association study.

## Author Contributions

AD: Designed experiments, analyzed the data, and wrote the paper. MR and AB: performed experiments and analyzed the data. AD, FC, MF, and CM-D: contributed writing the paper.

## Conflict of Interest Statement

The authors declare that the research was conducted in the absence of any commercial or financial relationships that could be construed as a potential conflict of interest.

## Footnotes

1 http://publiclines.versailles.inra.fr/
