# Supplementary material for: Quantitative Methods to Assess Differential Susceptibility of Arabidopsis thaliana Natural Accessions to Dickeya dadantii
Source: Front Plant Sci. 2017 Mar 28;8:394. doi: 10.3389/fpls.2017.00394 (PMC5368239; doi:10.3389/fpls.2017.00394)
Supplement: Supplementary file 1 [file Table_1.PDF]

Supplementary Table 1 : Mineral compostion of nutritional solution

	Solution 2 mM NO3-	Solution 10 mM NO3-
	**(mM)**	**(mM)**
KH_2PO_4	**0.25**	**0.25**
$MgSO_4$	**0.25**	**0.25**
KNO_3	**1.75**	**5**
CaN_2O_6	**0.125**	**2.5**
$CaCl_2$	**0.125**	**0**
NaCl	**0**	**0.2**
	**µM**	**µM**
$(NH_4)_6Mo_7O_{24}$	**0.04**	**0.04**
H_3BO_3	**24.3**	**24.3**
$MnSO_4$	**11.8**	**11.8**
$ZnSO_4$	**3.48**	**3.48**
Sequestrene 138 FE 100 Syngenta)	**0.001%**	**0.001%**
