# Supplementary material for: Quantitative Methods to Assess Differential Susceptibility of Arabidopsis thaliana Natural Accessions to Dickeya dadantii
Source: Front Plant Sci. 2017 Mar 28;8:394. doi: 10.3389/fpls.2017.00394 (PMC5368239; doi:10.3389/fpls.2017.00394)
Supplement: Supplementary file 2 [file Table_2.PDF]

Supplementary Table 2: Relationship between optical density of inoculum, bacterial density and theoratical bacterial DNA in inoculated leaves. The conversion was based on the following rules. A D. dadantii bacterial suspension of 0D600 =0.1 contains about 5x108 C.F.U./mL. A bacterial cell contains about 5 fg of DNA.

Inoculum	nb bacteria/leaf	Theoretical DNA (µg)/leaf
5 µL OD600=0.1	2,50E+06	0.013
5 µL OD600=0.2	5,00E+06	0.025
5 µL OD600=0.4	5,00E+07	0.05
5 µL OD600=0.8	5,00E+08	0.1
5 µL OD600=1	1,25E+08	0.125
