# Supplementary material for: Quantitative Methods to Assess Differential Susceptibility of Arabidopsis thaliana Natural Accessions to Dickeya dadantii
Source: Front Plant Sci. 2017 Mar 28;8:394. doi: 10.3389/fpls.2017.00394 (PMC5368239; doi:10.3389/fpls.2017.00394)
Supplement: Supplementary file 3 [file Data_Sheet_1.PDF]

Supplementary File 1 : Sequence alignment of PelA and RpoB primers with *D. solani* genome sequence. Mismatches are highlighted in bold.

Alignment of *PelA* primers *D. dadantii* vs genome *D. solani (5' → 3')*

PelA-F *D. dadantii* :	CCG**C**AACGTCTACATCCA**A**A
	||| |||||||||||||| |
Matching Sequence in D. solani :	CCG**T**AACGTCTACATCCA**G**A

RpoB-F *D. dadantii* :	AATCGAAGGTTCCGG**G**AT**T**C
	||||||||||||||| || |
Matching sequence in D. solani ::	AATCGAAGGTTCCGG**T**AT**C**C

RpoB-R *D. dadantii* :	**G**CCGTTACGGATA**T**CGATGAG
	|||||||||||| |||||||
Matching sequence in D. solani ::	**A**CCGTTACGGATA**C**CGATGAG
